# Leisure time patterns of children with and without disabilities: a cross-sectional latent class analysis

**DOI:** 10.3389/fspor.2025.1712055

**Published:** 2026-01-06

**Authors:** Christian Møller-Skau, Catherine A. N. Lorentzen, Shahram Moradi, Lars Bauger

**Affiliations:** 1 Research Group for Disability and Inclusion, Faculty of Health and Social Sciences, Department of Health, Social and Welfare Studies, University of South-Eastern Norway, Porsgrunn, Norway; 2 Research Group for Health Promotion in Settings, Faculty of Health and Social Sciences, Department of Health, Social and Welfare Studies, University of South-Eastern Norway, Tønsberg, Norway

**Keywords:** children disability, leisure time activities, organised activities, latent class analyses (LCAs), self-report, adolescent disability

## Abstract

**Introduction:**

Leisure participation supports children's health, social inclusion, and well-being, yet children with disabilities (CWD) often face barriers to participate in organised and physically demanding activities. This study examined differences in leisure-time patterns between children with and without disabilities.

**Methods:**

Cross-sectional data from 6,049 Norwegian children aged 10–13 years were analysed. Leisure time was assessed across six domains using twenty-two indicators. Latent Class Analysis identified leisure-time profiles, and multinomial logistic regression examined associations between disability status and profile membership, adjusting for sociodemographic factors.

**Results:**

Five leisure profiles emerged: *Home-oriented* (21%), *Social-oriented* (14%), *Aesthetic-oriented* (20%), *Physically-oriented* (31%), and *Screen-oriented* (14%). In the adjusted model, and when comparing to children without disability and having the *physically-oriented* as the reference group, CWD were more likely to belong to the *Screen-* (OR = 2.41, 95% CI: 1.80–3.21) and *Social-oriented* (OR = 2.04, 95% CI: 1.52–2.74) profiles.

**Discussion:**

CWD were less likely to be in profiles characterised by organised and physical leisure activities and more likely to be in profiles dominated by screen-based and informal activities, indicating persistent barriers to inclusive leisure-time participation. These findings underscore the importance of developing strategies that promote accessible, organised, and physical activity-based leisure opportunities, such as sports, while also ensuring sustained access to inclusive informal and digital spaces, such as neighbourhood facilities and e-sports. These efforts would support the social lives and well-being of CWD.

## 1 Introduction

Participation in leisure activities, whether through organised sports, cultural activities, or informal play, offers children opportunities for belonging, mastery, and enjoyment (1, 2). Such engagement supports the development of social skills and identity (3), as well as contributes to better physical health and well-being (2, 4, 5). Leisure time is commonly understood as the voluntary time free from obligations such as school or work, and life-maintaining activities such as sleeping or eating, allowing individuals to choose activities freely (6, 7). It is therefore not merely recreational; it plays a formative role in shaping children's physical activity levels, peer relationships, mental health, and long-term life satisfaction (2, 8–10). To emphasise its importance, leisure time is embedded as a right for all children in the United Nations Convention on the Rights of the Child (UNCRC) [(11), art. 31] and in the United Nations Human Rights [(12), art. 24]. The right to participate on equal terms with others in leisure is also embedded as a principle in Article 30 in the Convention on the Rights of Persons with Disabilities (CRPD) (13).

Children and youth leisure time in industrialised countries is often dominated by organised sports, digital leisure (e.g., gaming, social media, streaming), and cultural pursuits such as music and theatre (2, 14). While this pattern is broadly similar across European countries, Canada and Australia, national differences in economic conditions, cultural traditions, and policy frameworks shape how leisure time is allocated (1, 14). In Norway, where this study is conducted, organised sports plays a central role in children's leisure activities (15, 16). In addition, Norwegian children frequently engage in unstructured outdoor play in natural environments, home-based creative activities, and cultural practices such as music, theatre, and dance (10, 17, 18). This reflects both the country's nature-based tradition and its strong volunteer sector (19). In recent years, digital leisure, including gaming, social media, and streaming services, has become an increasingly dominating part of leisure among Norwegian children (18, 20), mirroring global trends (21, 22).

Inclusion is a key objective in Norway's welfare state (23), reflected in sectors like organised sports (24) and cultural activities (25). For example, the Norwegian Olympic and Paralympic Committee and Confederation of Sports (NIF) (26) embodies this through its slogan “Joy of Sport—for All”, which emphasises universal design and mainstreaming leisure activities to ensure equal opportunities, either through adapted mainstream activities or dedicated parasport programs (16, 24, 25). However, comprehensive data on children with disabilities (CWD) participation in mainstream sports (16), and, to the best of our knowledge, in the broader cultural sector is lacking. This absence of data limits the ability to evaluate inclusion efforts and identify barriers to CWD participation in organised leisure activities. How children spend their leisure time is shaped by several factors, including gender (2), socioeconomic background, place of residence, and disabilities (17, 27, 28). Research consistently demonstrates that CWD, compared to their peers without disability, are less likely to participate in organised leisure activities (17, 28, 29), and more likely to engage in unstructured activities (30) or home-based engagements, such as playing video games (31, 32). Furthermore, CWD typically participate in a narrower range of activities and experience fewer opportunities for social interaction (33), spending less time with friends (32, 34, 35). Also, CWD are less likely to be physically active than their peers without disability (36, 37). Differences also apply to digital arenas, where CWD report lower levels of social connections compared to children without disability, including on social media (38). Additionally, CWD tend to spend more time on screens overall (37). These patterns suggest that despite formal recognition of their rights, CWD face barriers to inclusive and meaningful leisure participation.

The prevalence of disability among children under 18 years remains somewhat uncertain due to differences in measurement and definitions (39). European estimates range from 4 to 8% (40, 41), while Norwegian estimates range from 2 to 10% (42, 43). Despite these uncertainties, CWD constitute a significant portion of the child population. Their participation in leisure activities is more strongly influenced by environmental barriers, family background and circumstances, and personal characteristics (4, 44–46) compared to their peers without disability (17, 18). Barriers such as the absence of elevators or wheelchair ramps, the competitive nature of certain activities, limited family income, and long travel distances can restrict access and participation in some leisure activities. In contrast, home-based activities, particularly e-sports and gaming that involve physical movement (e.g., Wii Sports, PS Move), have been identified as facilitators of participation (4, 45). Personal factors, including a child's physical appearance and reduced self-esteem, often stemming from self-perceived ability gaps compared to peers without disability, can also hinder participation (45). Although leisure time is frequently regarded as a space for inclusion and belonging (47), it may also serve as a site of exclusion and stigma. Negative experiences such as peer rejection, lack of accommodations, or societal attitudes that reinforce stereotypes can contribute to a diminished self-image and reduced participation (48). Taken together, these findings highlight the interplay between societal, familial, and individual factors that influence leisure participation among CWD.

Although numerous studies across high income-countries have examined leisure participation among CWD, the majority have focused on specific diagnoses (e.g., cerebral palsy, developmental disability) (35, 37, 49), particular types of leisure activities (e.g., physical activity, cultural engagement) (37, 50, 51), or barriers and facilitators to participation (4, 44, 45). Consequently, less is known about the overall patterns of leisure engagement when considering a broad range of activities, and how this compares to children without disabilities. Research that primarily targets specific disability types often fails to capture the diverse and heterogeneous experiences of CWD as a whole. This gap in the literature limits the potential to design inclusive policies and implement effective interventions that address the broader needs of this group.

To our knowledge, no studies have comprehensively addressed this topic using self-reported data and a person-centred approach, such as Latent Class Analysis (LCA), to capture the diversity of leisure-time profiles within a large, representative sample of children. Unlike variable-centred approaches that examine relationships between variables across the whole sample, LCA identifies subgroups of individuals with similar response patterns (52, 53). LCA is particularly suited for the purpose of this study, as it enables the identification of distinct leisure-time profiles rather than focusing on single activities in isolation. To address the observed gap in the literature, the present study identifies and compares leisure time participation patterns among children with and without disability. Specifically, we aim to develop a typology of leisure-time profiles and examine whether CWD differ from their peers without disability in their likelihood of belonging to these profiles.

## 2 Method

### 2.1 Description of the study and participants

This study uses cross-sectional data from the Ungdata Pluss project (54) collected in spring 2023. The data were gathered through an electronic survey conducted during school hours, which was based on the established Ungdata surveys (55) supplemented with additional questions. Children in fifth to seventh grades (aged 10–13) from 21 municipalities across two counties in the south-eastern part of Norway were invited to participate. Of the 11 848 children invited, 6 049 completed the survey, representing 51% of the total population.

### 2.2 Measures

#### 2.2.1 Leisure time

Leisure time is a complex, multidimensional phenomenon encompassing various aspects of individual behaviour, social interaction, and environmental context (5, 33). Leisure time was assessed using twenty-two questions, representing six leisure domains (See Table 1). In LCA, it is common practice to collapse response options into a smaller set for both theoretical and practical reasons, ensuring better interpretability (53). Therefore, some response options were collapsed due to small cell sizes and only slight variation in the responses (See Supplementary Table S1a,b for full operationalisation details). For instance, the question “How often do you do any of the following things close to where you live?—*Hang out on your own or with friends/siblings without doing anything special?*” originally included six response categories: “Rarely or never”,“A few times a month”, “About once a week”, “2–3 times a week”, “4–5 times a week”, and “About every day”. We recoded them into four categories: (1) “Rarely or never” and “A few times a month” were combined into “Rarely or never”; (2) “About once a week” remained unchanged; (3) “Between 2 and 3 times a week” remained unchanged; and (4) “4–5 times a week” and “About every day” were combined into “Four times a week or more”.

**Table 1:** Leisure questions.

Questions	Response
Home and neighbourhood	
*How often do you do any of the following things close to where you live?*	Rarely or never
1. Hang out on your own or with friends/siblings without doing anything special	About once a week
2. Play outside on your own or with friends/siblings (for example, playing football, cycling or playing tag)	Four times a week or moreBetween 2 and 3 times a week
*When you're at home, how often do you usually do the following things?*	
3. Have a visit from friends	Rarely or never
4. Do something nice with the adults in your family	About once a week
5. Help with housework (for example, cooking, cleaning, washing, hoovering, gardening)	Between 2 and 3 times a week
6. Make something or do things with your hands (for example drawing, painting, carpentry, handiwork or “tinkering”)	Four times a week or more
Digital leisure	
*Think about an ordinary day after school. Roughly how many hours do you spend on the following?*	1 h or less
7. Watch TV/films/series/YouTube, 8. Play computer games/TV games, 9. Play on a mobile phone/tablet, 10. On social media	Between 1 and 3 h
	3 h or more
	Less than once a week
	Once a week
	Several times a week
Organised leisure	
*What leisure activities do you do, and how often do you do them? One tick for each line.*	Never
11. Sports, 12. Dance or ballet, 13. Theatre, singing or music, 14. Youth club or junior club, 15. Other activities	Less than once a week
	Once a week
	Several times a week
Unorganised physically active leisure	
16. How often do you normally work out?	Never
	Once a week or less
	2–5 times a week
	Every day
17. How often do you do outdoor activities in the summer—such as hiking or cycling in the great outdoors, swimming, fishing or sleeping in the wild?	Never
	Rarely or sometimes
	Often or very often
18. How often do you do outdoor activities in the winter—such as skiing, snowboarding or ice skating?	
School-based leisure	
19. How much time do you usually spend on schoolwork during the afternoon and evening?	No time
	30 min or less
	Between 30 min and 1 h
	2 h or more
Schoolwork and unorganised social or academic leisure	
*How often do you usually?*	Rarely or never
20. Go to a café, shopping centre or shops?	Once a week
	Between 2 and 3 times a week
	Four times a week or more
*How often do you usuall?*	Rarely or never
21. Go to the library?	Once a week or more
*Think about an ordinary day after school. Roughly how many hours do you spend on?*	Never
22. Reading books, comics or listening to audiobooks?	1 h or less
	More than 1 h

Note. All questions are marked with numbers. Text in italic is lead-in statement for the question that follows.

Supplementary Table S1 provides a complete overview of the original response options and their collapsed categories.

#### 2.2.2 Disability

Disability was conceptualised according to the relational model, which means disability is understood as the result of interactions between individual limitations and environmental expectations that do not align with individual capabilities (56, 57). This perspective highlights the importance of addressing both societal barriers and individual capabilities to ensure meaningful and equitable access to leisure opportunities.

Children self-reported functional limitations in three physical (seeing, hearing, and moving) and three cognitive domains (reading or writing, understanding, and concentrating), with response options: “No, never”, “Yes, sometimes”, “Yes, often”, and “Yes, all the time” (58, 59). Those who reported limitations “Yes, often” or “Yes, all the time” were subsequently asked whether these limitations hindered their participation in school or leisure activities, using the same response categories. Following recommended practice (60–62), we applied the “Often or more” cut-off to identify children as having a disability if they reported a limitation and a corresponding hindrance in either domain at the level of “often” or “all the time”. This two-step operationalisation was based on a context-sensitive definition of disability in line with the relational model of disability, reflecting both individual impairments and perceived environmental barriers (62).

#### 2.2.3 Socio-demographic background

Sex was recorded as a binary variable (*Boy/Girl*), and Grade level encompassed either *5th, 6th, or 7th grade*. The language spoken at home was used to identify immigrant background, categorised into two groups: 1) those who reported speaking *Only Norwegian* at home, and 2) those who reported speaking either both Norwegian and another language at home or merely another language than Norwegian, labelled *Norwegian and/or another language*. Centrality was derived from administrative records and classified according to Statistics Norway's centrality index (63). Municipalities were grouped into two categories: *Urban*, representing the most central areas, and *Rural*, representing both mid-central and the least central areas.

Socioeconomic status (SES) was measured using four items from the Family Affluence Scale (FAS) (64)*: Does your family have a car?, Do you have your own bedroom?, How many times did you go abroad on holiday last year?* and *How many computers and tablets does your family have*?. A mean score was computed to represent the total family resources, ranging from 0 (lowest) to 3 (highest). This score was then transformed into a categorical variable by dividing it into three equal groups (tertiles) based on its distribution (65): *Low* (lowest third of the distribution), *Medium* (middle third of the distribution), and *High* (highest third of the distribution).

#### 2.2.4 Survey validation

The questionnaire was validated through cognitive interviews with children from both mainstream classes and special education classes (58). Additionally, the survey underwent pilot testing to ensure clarity and suitability for all children (59, 66).

### 2.3 Analytic strategy

All statistical analyses were performed using the software R (version 2023.03.1). The sample size varied slightly across analyses due to missing responses on individual variables.

#### 2.3.1 Descriptive analysis

Socio-demographic characteristics of the overall sample, and stratified by disability status, were assessed through sub-sample n's and proportions. Differences between children with and without disability were assessed through Pearson's Chi-square tests. The significance threshold was set at *p* < .05.

#### 2.3.2 Latent Class Analysis

LCA is a statistical method used to identify unobserved, heterogeneous subgroups or “latent classes” within a population, based on patterns of responses to a chosen set of observed variables (67, 68). We used the poLCA package for R (69, 70). Both an empirical (Information Criteria) and a practical criterion (interpretability) were used to determine the optimal number of classes [(53, p. 292]. Both the Bayesian Information Criterion (BIC), sample-size adjusted BIC (SABIC), Akaike Information Criterion (AIC), Consistent Akaike Information Criterion (CAIC), as well as the Lo–Mendell–Rubin likelihood ratio test, and practical considerations (interpretability and no class smaller than 5%) (53, 71) were considered. However, given our large sample size, we followed the recommendations to use the BIC as it tends to indicate a better fit than the AIC (72). Classification quality was assessed using entropy, the average maximum posterior probability, and the average posterior probabilities by assigned class (AvePP).

After selecting the class solution best fitted by the criterions, participants were assigned to their most likely class based on posterior probabilities. This approach, commonly referred to as the “classify-then-analyse” method, is widely used in applied LCA research because it is simple and interpretable (53, 73, 74). Lastly, we qualitatively examined any patterns within the classes and assigned names that would be meaningful for describing them.

#### 2.3.3 Covariate analysis

Associations between disability status and class membership were examined using a Chi-squared test to compare the proportions of children with and without disabilities across the latent classes. We used a Bonferroni correction to control for multiple comparisons. We then estimated two multinomial logistic regression models, using Class 4 (Physically-oriented) as the reference category for the dependent variable (Latent class) and “Not disabled” as the reference group for the independent variable (Disability status). Class 4 was chosen as the reference class because it included the largest proportion of children and the lowest proportion of CWD (see the supplementary for analyses using the other classes as reference). Model 1 included only disability status as independent variable, while Model 2 adjusted for sex, grade, language spoken at home, centrality and SES. Model fit was assessed using McFadden's pseudo *R*^2^ (75), AIC and BIC. We report odds ratios (OR) with 95% confidence intervals (65).

### 2.4 Ethics

This study is approved by the Regional Committee for Medical and Health Research Ethics Norway (2024/721601), and the Norwegian Agency for Shared Services in Education and Research (372074). Prior to the survey, all participants were informed that participation was voluntary. Digital informed consent was obtained from the guardians of all participating children. To promote inclusion, the Ungdata Pluss data collection adhered to principles of universal design, ensuring readability, appropriate contrast, font size, and integrated audio support for all questions. Also, schools were encouraged to provide flexible arrangements such as extra time and separate rooms (59, 66).

## 3 Results

Descriptive statistics for the sample are presented in Table 2. CWD represented 7.9% of the sample. The sex distribution was equal across the sample. Grade level distribution was also consistent, with approximately one-third of children in each of the three grades. One in five children reported speaking a language other than Norwegian at home. A significant difference was observed between CWD and those without disabilities in terms of language spoken at home: 26% of CWD spoke *Norwegian and/or another language* at home, compared to 20% of their peers without disability (*p* *<* *.05*). The majority of children resided in urban areas, with no notable differences between groups. No notable difference was found in socioeconomic status between the groups.

**Table 2:** Descriptive analysis.

Variables	Overall	Not Disabled	Disabled	*p*-value^a^
	(*N* = 6,001–6,049)	(*n* = 5,034–5,062)	(*n* = 472–476)	
	% (n)	% (n)	% (n)	
Sex				.3
Boy	51 (3,050)	51 (2,565)	48 (228)	
Girl	49 (2,997)	49 (2,480)	52 (245)	
Grade level				.7
5th	33 (1,996)	32 (1,639)	33 (155)	
6th	34 (2,055)	34 (1,738)	33 (154)	
7th	33 (1,986)	33 (1,677)	35 (164)	
Language spoken at home				.004*
Only Norwegian	79 (4,770)	80 (4,022)	74 (351)	
Norwegian and/or another language	21 (1,231)	20 (1,012)	26 (121)	
Place of residence				.6
Rural	16 (988)	16 (833)	16 (74)	
Urban	84 (5,061)	84 (4,229)	84 (402)	
Socioeconomic status				.061
Low	33 (2,013)	33 (1,653)	38 (180)	
Medium	33 (2,012)	34 (1,711)	30 (143)	
High	33 (2,012)	34 (1,698)	32 (153)	

a Pearson's Chi-squared test.

* Significant at *p* < .005.

### 3.1 Model selection by latent class analysis

**Figure 1:**
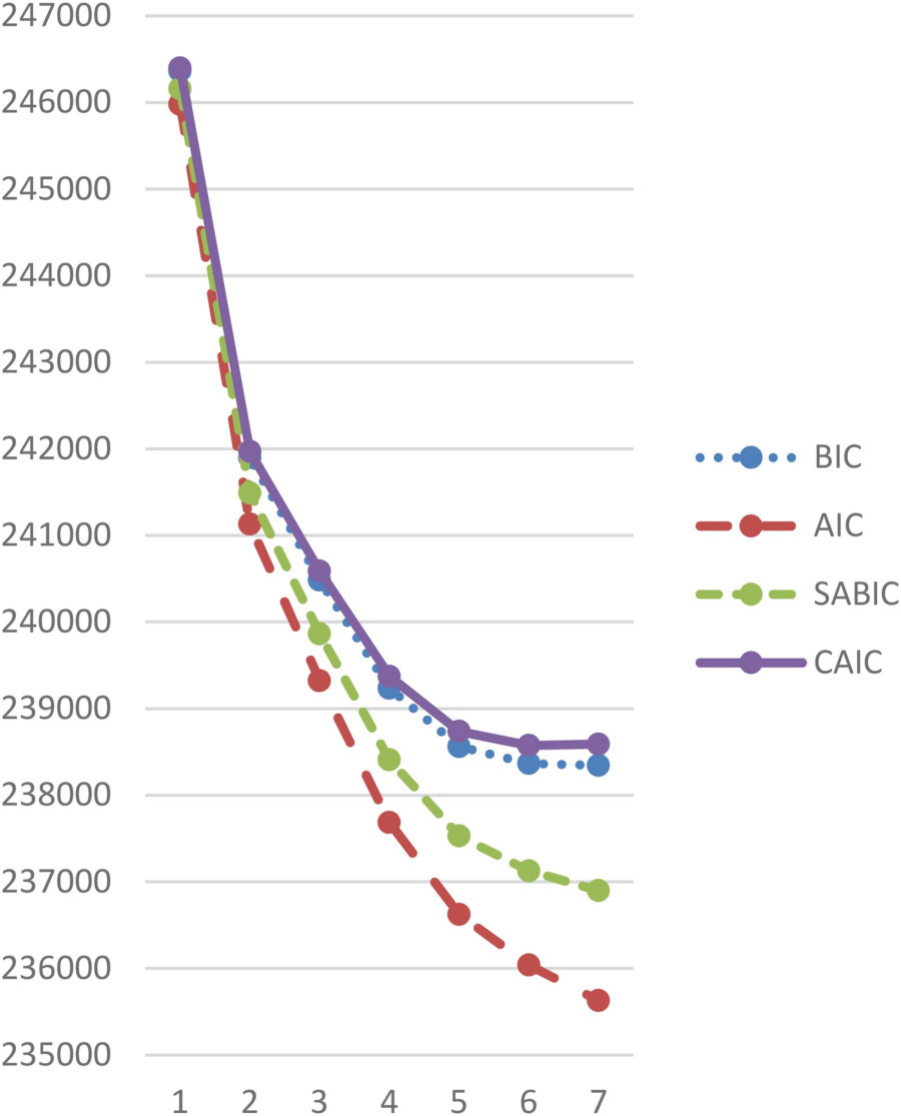
IC-criterion plot.

To determine the optimal number of latent classes, we compared model fit statistics across solutions ranging from one to seven classes (see Table 3). The Lo-Mendell-Rubin (Lrt) test was statistically significant (*p* < .001) for all models, supporting the addition of classes up to seven, indicating a good fit (71). As Table 3 shows, the five-class model showed the most favourable balance between empirical fit and interpretability and avoided very small classes. Specifically, the BIC and SABIC exhibited a clear “elbow” at five classes (see Figure 1), indicating a point of diminishing returns in model improvement (53, 71). Additionally, the five-class solution revealed that the smallest class comprised 14.3% of the sample, well above the recommended minimum of 5% to ensure sufficient representation and analytical stability (71, 72).

**Table 3:** Information criterion for LCA analysis.

Class	Log likelihood	BIC	AIC	SABIC	CAIC	Lrt	Smallest class (%)
1	−122,932.9	246,362.1	245,979.7	246,158.3	246,396.4	–	–
2	−120,450.1	241,901.5	241,130.1	241,490.5	241,970.9	4,965.603**	42.3
3	−119,488.9	240,484.2	239,323.8	239,865.9	240,588.6	1,922.304**	23.8
4	−118,611.5	239,234.4	237,684.9	238,408.7	239,373.7	1,754.903**	15.5
5	**−118,023.1**	**238,562.8**	**236**,**624.2**	**237,529.8**	**238,737.1**	**1,176.67** **	**14.3**
6	−117,672.4	238,366.4	236,038.9	237,126.1	238,575.8	701.367**	10.3
7	−117v409.2	238,345.1	235,628.5	236,897.5	238,589.4	526.391**	8.6

BIC, Bayesian Information Criterion; SABIC, sample-size adjusted BIC; AIC, Akaike Information Criterion; CAIC, Consistent Akaike Information Criterion, Lrt, Lo-Medell-Rubin test.

* *p* <  .005.

** *p*  < .001.

Further on, the entropy for the five-class model was 0.69, indicating moderate classification certainty (74, 76, 77). The average maximum posterior probability was 0.81 (71.3% ≥ 0.70; 59.8% ≥ 0.80; 44.3% ≥ 0.90). AvePP were high (see Supplementary Figure S1): Class 1 = 0.80, Class 2 = 0.81, Class 3 = 0.79, Class 4 = 0.80, Class 5 = 0.83, suggesting acceptable though not perfect, separation of classes (76). The largest cross-classification occurred between Class 3 and Class 5, as well as between Class 1 and Class 4.

As a result, we chose to follow the five-class solution, given both the statistical fit criteria and the fact that classes were meaningfully interpretable (53, 71, 76).

#### 3.1.1 Characteristics of the identified latent classes

Tables 4a–e present descriptive statistics for the five latent classes. Although these classes represent varied patterns of leisure engagement, each class is characterised by a dominant type of activity. It is worth noting that belonging to one class, dominated by one type of leisure engagement, does not mean that the participants do not engage in other leisure activities. Below is a qualitative description of each class, highlighting some key characteristics for each group.

**Table 4a:** Home and neighbourhood leisure.

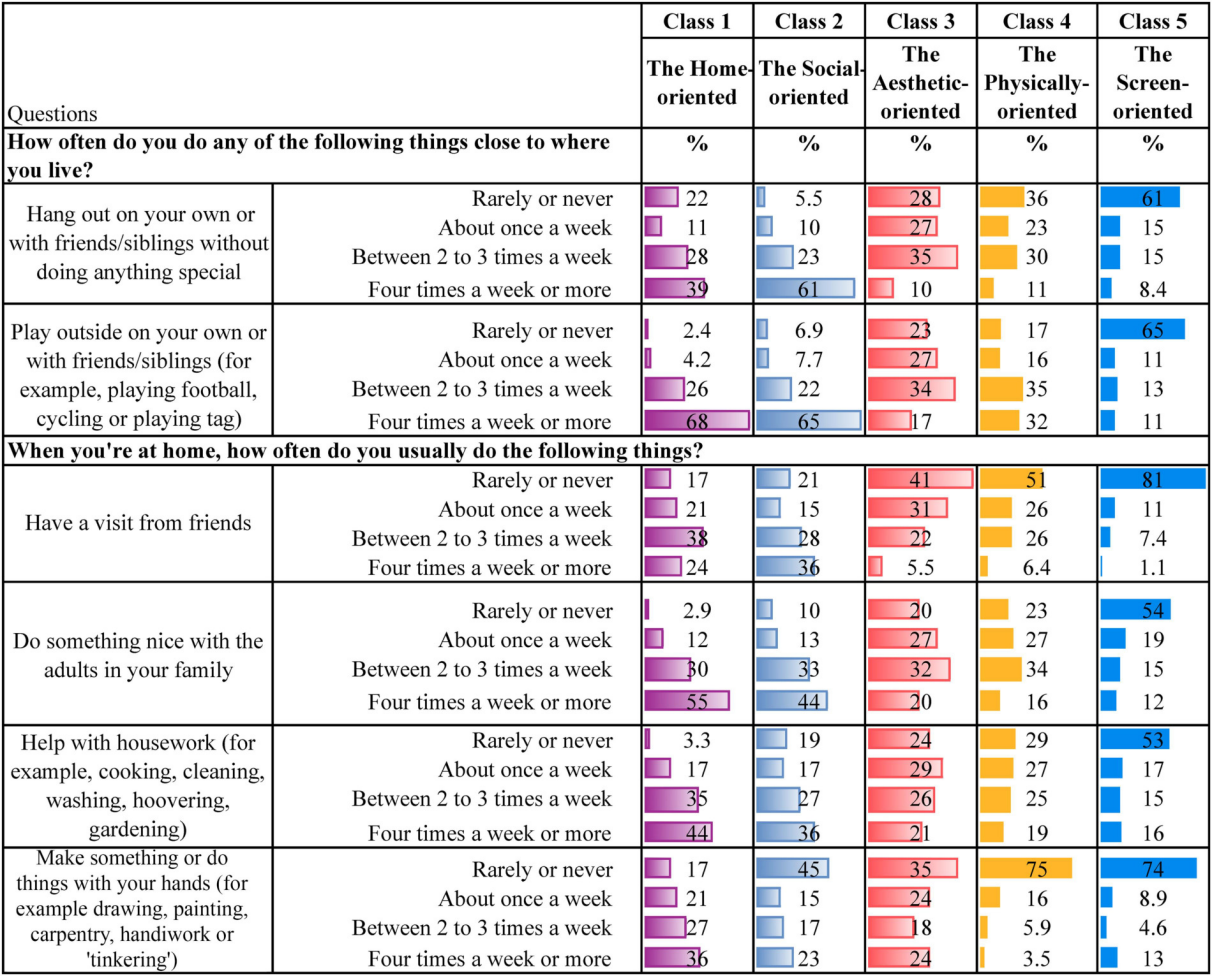

**Table 4b:** Digital leisure.

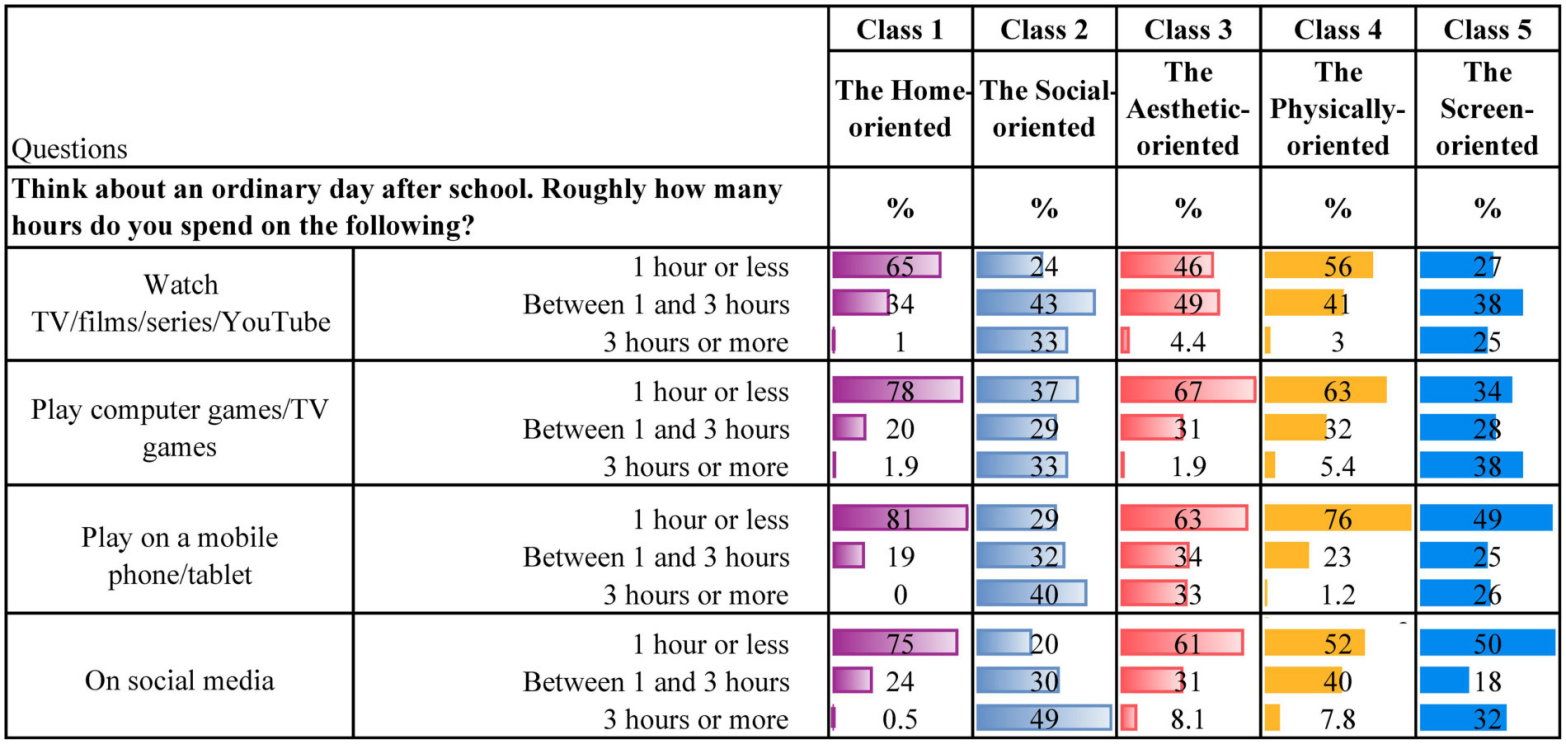

**Table 4c:** Organised leisure.

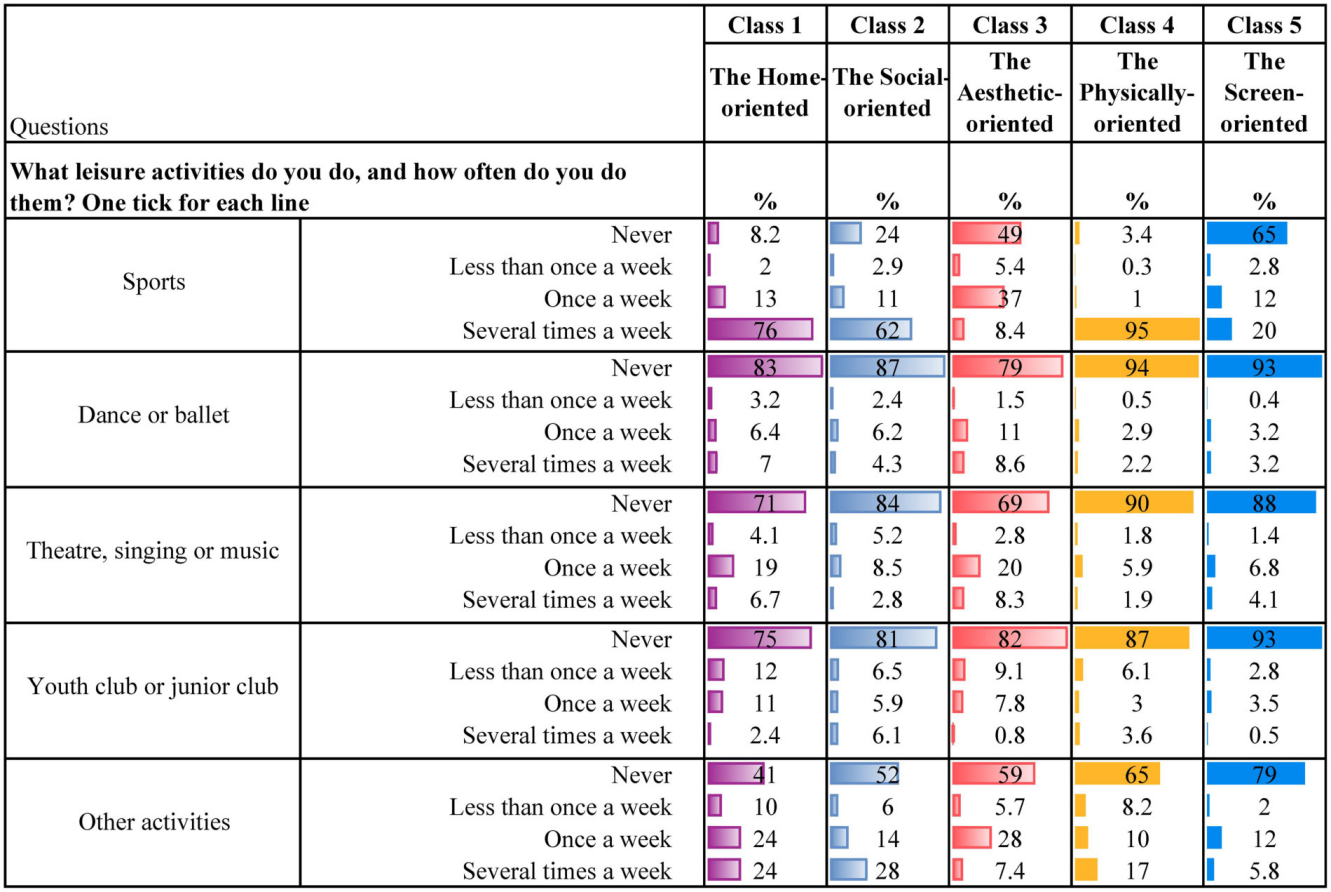

**Table 4d:** Unorganised physically active leisure.

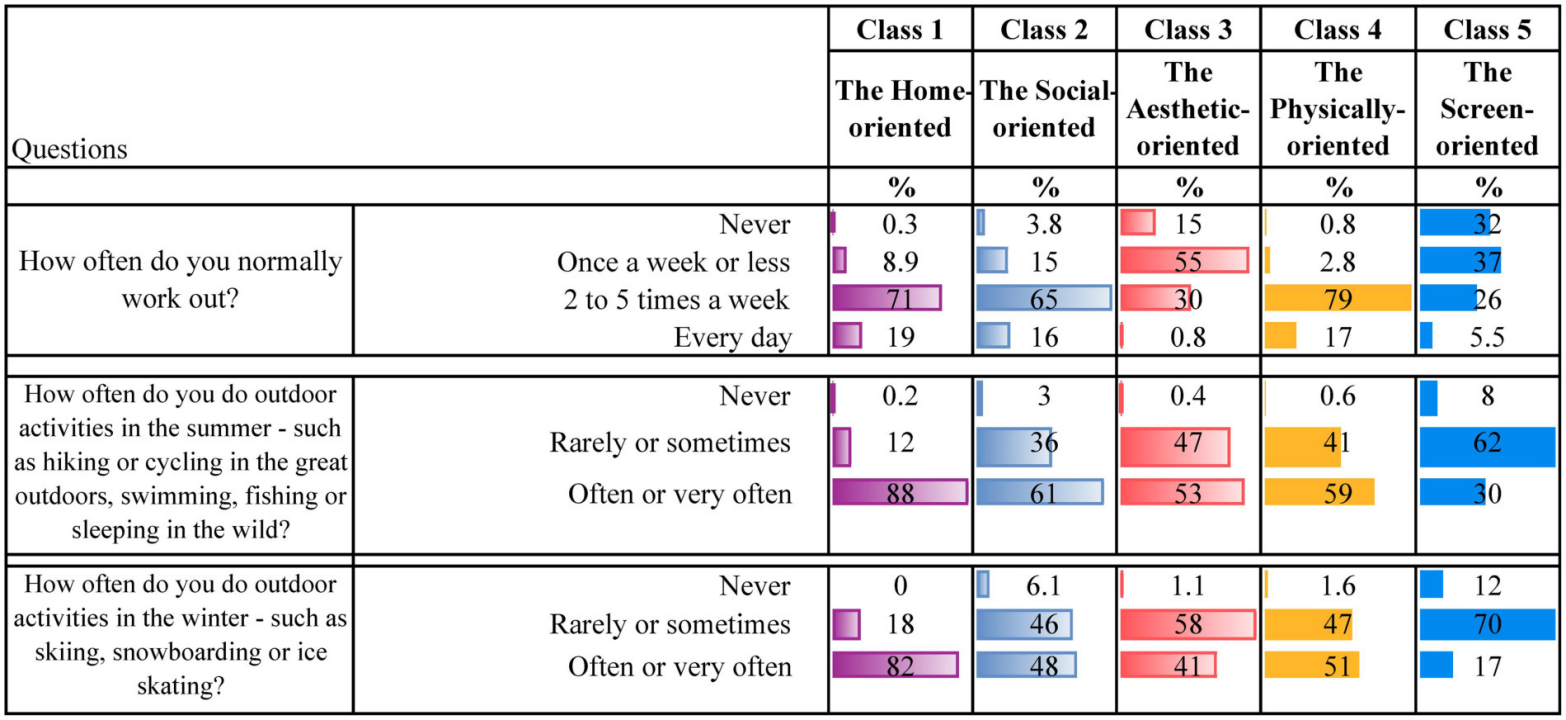

**Table 4e:** Schoolwork and unorganised social or academic leisure.

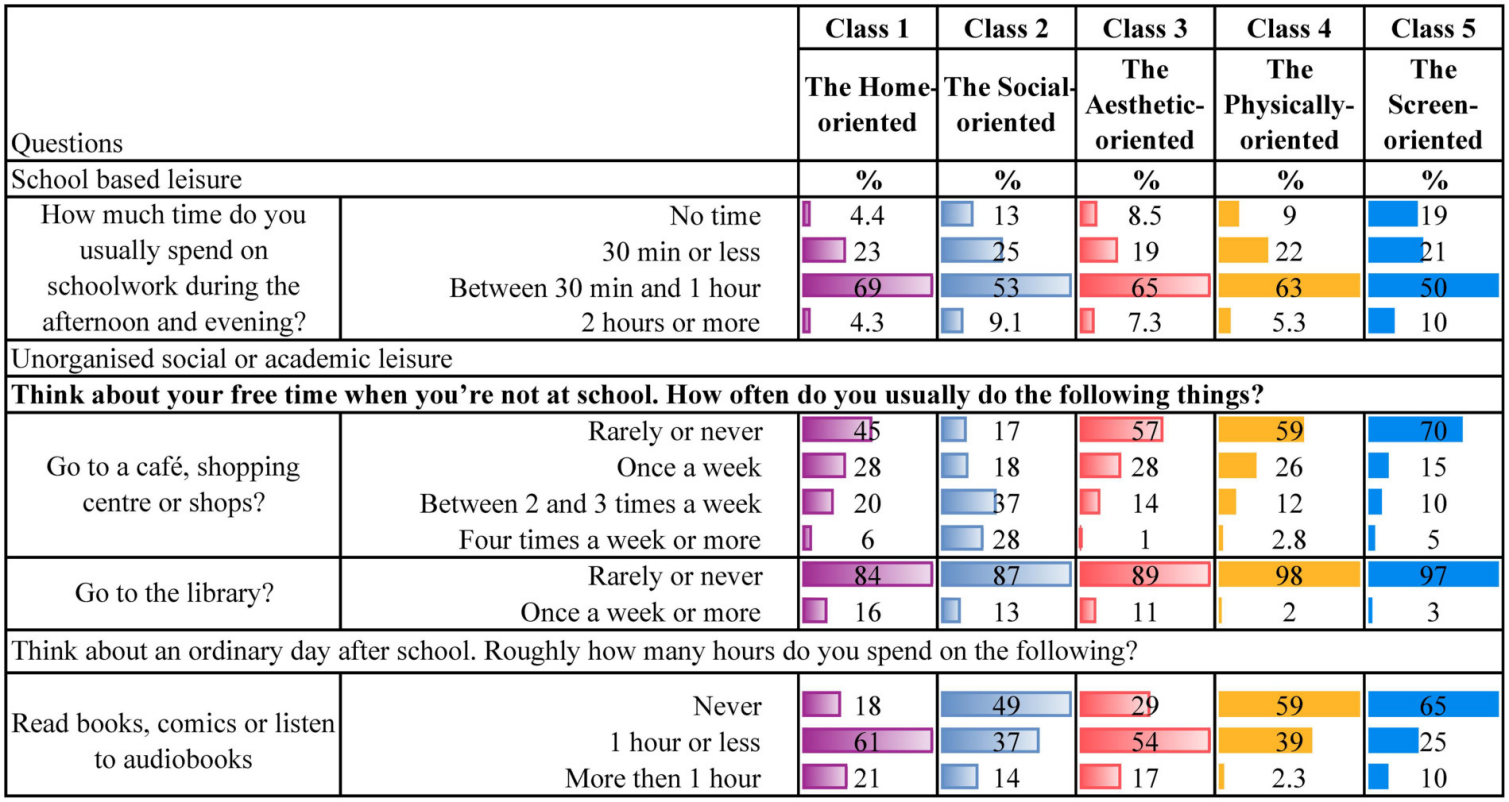

**Class 1, the Home-oriented** (*n* = 1,280, 21%), is characterised by high engagement in leisure activities at home or in the neighbourhood. Individuals in this class tend to excel in activities such as spending time with friends and family at home, regularly contributing to household tasks, and engaging in crafts within their own home environment. Additionally, they are among those with the most time spent reading books/comics or listening to audiobooks. They are also physically active and report the highest engagement in unorganised nature-based leisure activities during both summer and winter compared to the other classes. Also, they are the group who reports the second highest participation in organised sports. They regularly visit cafes, shopping centres and the library. Their screen time after school is low, and their social media use is minimal.

**Class 2, the Social-oriented** (*n* = 844, 14%), is characterised by particularly high engagement in activities involving social interactions, such as spending time with family, visiting friends and going shopping. They are the class that spends the most time on social media compared to the other classes. In addition, they spend considerable time watching YouTube, movies and playing video games. However, they are also relatively physically active, participating in sports several times a week, working out regularly, and engaging in unorganised nature-based activities during the summer and winter.

**Class 3, the Aesthetic-oriented** (*n* = 1,185, 20%), is characterised by the highest engagement in organised artistic activities (ballet, dance, theatre, singing, and music) and doing crafts at home. Additionally, individuals in this class spend more time reading books/comics or listening to audiobooks compared to most other groups. They occasionally have friends over to visit, though less frequently than children in the other classes. Additionally, they also engage in other activities in the home or neighbourhood environment. Their social media use is low, but they are average users of other screen-related activities. Also, they work out regularly and occasionally participate in unorganised nature-based activities during both summer and winter.

**Class 4, the Physically-oriented** (*n* = 1,902, 31%), is characterised by the highest level of participation in organised sports activities, with class members engaging in these several times a week. They also frequently take part in unorganised nature-based activities and often play outside their homes. However, they rarely have friends over or engage in activities at home. Their screen time is average, but they almost never read books or listen to audiobooks. Additionally, they seldom visit cafes, shopping centres, or libraries.

**Class 5, the Screen-oriented** (*n* = 838, 14%), is characterised by below-average involvement in most leisure activities but above-average screen time. Individuals in this class spend a significant amount of time both watching TV/films, playing video games and using social media. They rarely play or engage in activities within their neighbourhood and almost never have friends over at home. They are minimally involved in organised activities but occasionally work out. They engage less in unorganised nature-based leisure activities than their peers. They seldom, if ever, go to cafés or shopping centres and do not visit libraries. Lastly, they are among those spending the least amount of time reading books/comics and listening to audiobooks.

As initially described, there are overlaps between the classes, which is common in LCA (71, 74). For instance, members of both the *Home-* and the *Physically-oriented* groups participate in organised sports several times a week. Those belonging to the *Social-oriented* group also participate in sports but are more engaged in informal activities such as “hanging out” with friends and engaging in informal outdoor play. Both the *Social-* and *Screen-oriented* groups report high levels of screen time; however, the key distinction is that the *Screen-oriented* group shows minimal engagement in other leisure domains. In contrast, the *Social-oriented* group strikes a balance between digital activity and physical and social participation. The *Aesthetic-oriented* group stands out for its strong involvement in artistic activities, including dance, music, and crafts. The *Physically-oriented* group reports relatively low screen time and engages frequently in organised sports, whereas the *Screen-oriented* group is less physically active than all other classes.

### 3.2 Distribution of children with and without disabilities in the latent classes

Table 5 presents the distribution of children with and without disability across the five latent leisure profiles (*n* = 5,538). Among CWD, the highest proportion was in the *Screen-oriented* class (23%). In contrast, the smallest proportion of children without disability (13%) belonged to this class. The *Physically-oriented* class was the most common profile among children without disability (33%), but 22% of CWD were also in this class.

**Table 5:** Disability status by leisure time profiles.

Latent Class	Not Disabled %	Disabled %
Class 1 The Home-oriented	21	15
Class 2 The Social-oriented	14	20
Class 3 The Aesthetic-oriented	20	19
Class 4 The Physically-oriented	33	22
Class 5 The Screen-oriented	13	23

*n* = 5,538.

*χ*^2^(4, 5,538) = 64.26 *p* < 001, Cramer's V = 0.1.

The chi-square test revealed a statistically significant association between latent class membership and disability status, with a small effect size (Cramer's V = .01, *p* *<* .001). After Bonferroni correction, the following pairwise comparisons were statistically significant: Class 1 vs. Class 2, Class 1 vs. Class 5, Class 2 vs. Class 4, Class 3 vs. Class 5 and Class 4 vs. Class 5.

### 3.3 Multinomial logistic regression analysis

As shown in Table 6, Model 1 explained little of the variation in the class membership (McFadden's R² of 0.08), while Model 2 showed modest improvement (McFadden's *R*^2^ of 0.13). AIC and BIC values were also lower for the adjusted model, supporting a better fit when sociodemographic covariates were included.

**Table 6:** Multinomial regression of latent class analysis with class 4 (Physically-oriented) as the reference class.

Models	**The Home-oriented (Class 1)** OR (95% CI)	**The Social-oriented (Class 2)** OR (95% CI)	**The Aesthetic-oriented (Class 3)** OR (95% CI)	**The Screen-oriented (Class 5)** OR (95% CI)
**Model 1** (*n* = 5,538)
Disability status (ref. Not disabled)				
Disabled	1.08 (0.80–1.47)	2.14** (1.60–2.86)	1.40* (1.05–1.87)	2.57** (1.94–3.41)
**Model 2** (*n* = 5,476)				
Disability status (ref. Not disabled)				
Disabled	1.08 (0.79–1.47)	2.04** (1.52–2.74)	1.31 (0.97–1.76)	2.41** (1.80–3.21)
Sex (ref. Boy)				
Girls	1.84** (1.58–2.15)	1.23* (1.04–1.46)	2.93** (2.50–3.44)	1.01 (0.85–1.21)
Grade (ref. 5th)				
6th	0.63** (0.52–0.75)	0.83 (0.67–1.02)	0.71** (0.59–0.87)	0.90 (0.72–1.13)
7th	0.34** (0.28–0.41)	0.61** (0.49–0.75)	0.58** (0.48–0.70)	0.98 (0.79–1.21)
Language spoken at home (ref. Only Norwegian)				
Norwegian and another language	0.59** (0.51–0.69)	2.03** (1.66–2.49)	1.39** (1.13–1.69)	1.50** (1.21–1.86)
Centrality (ref. Rural)				
Urban	0.59** (0.51–0.69)	0.78* (0.66–0.93)	0.64** (0.55–0.74)	0.86 (0.72–1.03)
SES (ref. Low)				
Medium	1.24* (1.08–1.42)	1.18* (1.02–1.37)	0.78** (0.68–0.89)	0.56** (0.48–0.65)
High	1.09 (0.95–1.24)	1.18* (1.02–1.37)	1.16* (1.01–1.32)	1.18* (1.01–1.37)

* *p* < .05.

** *p* < 001.

Model 1: McFadden *R*^2^ 0.08, AIC 17,232.14, BIC 17,285.1.

Model 2: McFadden R^2^ 0.13, AIC 16,489.45, BIC 16,727.5.

Model 1 shows that CWD were more than twice as likely as children without disability to belong to the *Social-* and *Screen-oriented* classes compared to the *Physically-oriented* class, which was used as the reference group. They were also 40% more likely to belong to the *Aesthetic-oriented* class, while no significant difference was observed for the *Home-oriented* class. After adjusting for sociodemographic variables (Model 2), the difference regarding the *Social-* and *Screen-oriented* classes remained largely unchanged. CWD were still significantly more likely than their peers without disability to belong to these classes compared to the *Physically-oriented* class. However, the association with the *Aesthetic-oriented* class was reduced (OR = 1.31) and was no longer significant. The *Home-oriented* class continued to show no significant differences.

## 4 Discussion

This study describes the differences in leisure-time profiles between children with and without disability through a person-centred approach. LCA identified five distinct (latent) classes: *Home-, Social-, Aesthetic-, Physically-* and *Screen-oriented.* While these profiles differ, the overall pattern aligns with previous research (18) and national reports (78) on Norwegian children's leisure time, indicating a similar diversity in children's leisure activities.

Our study further showed that CWD were more than twice as likely as children without disability to belong to the *Screen-* and the *Social-oriented* classes, when compared with the *Physically-oriented* class. Additionally, CWD were 30% more likely to be in the *Aesthetic-oriented* profile compared to their peers without disability. These results suggest that, compared to children without disability, CWD are more likely to belong to profiles where organised sports are less central, and informal- and screen-based activities are more present.

### 4.1 Physically-oriented profile

The *Physically-oriented* profile is characterised by a strong emphasis on organised sport activities and was the most prevalent among the overall population, as well as among children without disability. This is not surprising given the extensive cultural and institutional support for sports throughout childhood in Norway (17, 19, 79), and aligns with national reports indicating that participation in organised sports is a central part of Norwegian children's leisure time (10, 78). We found that CWD were underrepresented in this profile, a result consistent with well-documented disparities in participation in organised sports and physical activities between children with and without disability (29, 33, 38, 80, 81). These disparities are often attributed to barriers such as lack of accessibility, limited inclusion in activities, and social exclusion (4, 45, 79). Our findings therefore support earlier research, which shows that CWD are more likely to be engaged in activities that do not heavily involve physical activity or organised sports.

### 4.2 Screen-oriented profile

In contrast to the differences observed with the *Physically-oriented* profile*,* CWD were more likely than their peers without disability to belong to the *Screen-oriented* profile. This finding is consistent with previous research indicating higher levels of screen-based activity among CWD (38, 82, 83). For instance, Ng et al. (82) found that CWD in Europe spent significantly more time using screens compared to their peers without disability. Additionally, a systematic review by Vanderlo et al. (83) revealed that screen time was a predominant after-school activity for CWD worldwide.

On one hand, the high likelihood of CWD belonging to groups where screen time is a dominant part of leisure time raises concerns as screen time is often associated with sedentary behaviour and linked to adverse physical and mental health outcomes (84, 85). Also, Finnvold and Dokken (38) found that CWD report less inclusion in digital communities than their peers. On the other hand, while screen-based leisure is frequently viewed through a negative lens (82, 84), it can also serve as a meaningful and inclusive space for CWD, offering autonomy, creativity, and social connection (83, 86, 87), particularly for those who face physical, social, or environmental barriers in more organised offline leisure settings (45). For instance, Bolic Baric et al. (68) found that children with ADHD engaged more in online gaming and digital networking than their peers, but also benefited from these activities through skill development and social interaction. Gaming and e-sports might provide unique opportunities for inclusion, as they can often be played on equal terms regardless of disabilities (88, 89). For example, an individual in a wheelchair can perform just as well as a peer without disabilities in a digital soccer game. Additionally, video games have been shown to enhance learning, promote physical activity through active video games, and improve problem-solving skills (89).

It is essential to explore further how digital leisure can promote inclusion, social connection, and even physical activity for CWD. For instance, the rise of organised e-sports may provide alternative arenas for social participation (45, 68, 86, 90). At the same time, it is important to examine the quality and depth of social interactions facilitated through digital platforms. While digital communication can foster connection, it may also displace important, health-promoting face-to-face interaction (38). Maintaining a healthy balance between digital engagement and face-to-face interactions remains important for fostering overall well-being (91–93).

### 4.3 Social-oriented profile

Interestingly, CWD were also more likely to belong to the *Social-oriented* profile compared to their peers without disability. This profile is characterised by higher levels of physical activity, digital engagement, and in-person socialising, reflecting a group of children who are highly socially engaged both online and across multiple offline settings, including with friends and caregivers. For instance, children in this profile are the most active in playing with siblings and friends close to home. These findings are at odds with the assumption that CWD participate less in social activities than their peers (33, 35, 38). The greater likelihood of CWD belonging to this group might reflect the accessibility and inclusivity of informal, home-based social settings, which often allow for greater flexibility, individual adaptation, and emotional safety. These findings resonate with previous qualitative research by Melbø and Ytterhus (32), who found that having friends close to home enhances the social lives of CWD, expands their networks, and reduces experiences of bullying, as they have well-known people around them. These findings highlight the importance of the local neighbourhood for CWD, as it may serve as a community space where they can build meaningful relationships with peers (47).

### 4.4 Aesthetic-oriented profile

CWD were also somewhat more likely than their peers without disability to belong to the *Aesthetic-oriented* profile in the unadjusted model. However, this association attenuated and became non-significant after adjusting for sociodemographic variables.

While cultural activities are generally less common than organised sports among Norwegian children (17), they are also associated with better well-being, self-esteem and physical outcomes (94, 95). These activities are often less physically demanding, more individually adaptable, and may occur in more inclusive or supportive environments (96). Although the adjusted association was not statistically significant, the descriptive pattern aligns with previous research indicating that CWD tend to engage more in informal or individually adaptable activities and less in competitive or physically demanding ones (33, 45). Artistic activities in particular have shown to provide psychosocial benefits, including opportunities for self-expression, mastery, and identity development (94–96). These factors are particularly valuable for children who may experience exclusion in other domains of life.

### 4.5 Home-oriented profile

There was no difference between children with and without disabilities in the likelihood of belonging to the *Home-oriented* profile. This profile, although predominantly characterised by a high level of leisure pursuits within the home and neighbourhood environment, also includes relatively high levels of physical activity, with frequent participation in organised leisure activities and outdoor, nature-based activities during both summer and winter, alongside limited screen time. The fact that CWD were not more likely to belong to this group reinforces the broader pattern that CWD are more inclined to belong to profiles characterised by more informal activity, such as the *Social-* and *Screen-oriented* profiles.

### 4.6 Overall discussion

Overall, this study's findings, which show a higher likelihood of CWD belonging to leisure profiles dominated by informal activities, can be interpreted in the light of the relational model of disability. This model conceptualises disability as the outcome of interactions between individual functional limitations and environmental expectations (56, 57). In the Norwegian context, organised sports represent one of the most prominent forms of formal leisure activities (16, 78). However, as discussed, the lower participation of CWD in organised sports may reflect existing barriers within this setting (45, 97). Although Norway promotes inclusive sport policies (24, 25), several factors continue to hinder participation. For instance, organised sports are often rule-based, demand sustained attention and commitment and can be more “serious” in nature (19). These structural and cultural features, such as competitive norms and complex rules, can create significant barriers, which, when combined with individual functional limitations, further restrict participation for CWD. In contrast, informal leisure activities, such as digital and home-based activities, may reflect more flexible and inclusive environments, thereby mitigating the impact of environmental barriers. These findings underscore the importance of addressing not only physical accessibility but also social norms and prevailing attitudes. Creating equitable opportunities for leisure participation requires environments that are responsive to diverse needs and capable of accommodating individual differences and constraints. Our findings raise important questions for future research on how to enhance the leisure experiences of CWD. Should the focus be on strengthening their opportunities to participate in mainstream organised activities, such as sports and cultural programs, which dominate the leisure landscape for most Norwegian children? Or is there a greater need for tailored organised activities, such as para-sports, that better accommodate individual needs? Furthermore, what is the relative importance of informal leisure activities compared to organised options in shaping positive experiences for CWD?

The benefits of inclusive leisure settings can be substantial, including enhanced self-determination, friendship, and learning opportunities for CWD (98). Participation in mainstream sports or other organised leisure activities can enhance the feelings of mastery, inclusion, and belonging (28, 32). However, there is limited knowledge about those who do not experience inclusion in such settings. A report from the Norwegian (99) highlights instances where mainstream leisure activities failed to be inclusive due to prejudice that hindered participation. This illustrates the complexity of mainstream inclusion, where barriers are not only physical but also social, such as rules, norms, and, specifically in sports, a competitive culture (4, 45).

Furthermore, a report by Rambøll (97) argues that barriers to inclusive organised leisure stem from a lack of competence within sports clubs, as well as the inherent complexity of inclusion due to the uniqueness of each child with a disability. This uniqueness often requires tailored solutions to ensure that participation provides an opportunity for mastery (97). Therefore, tailored activities, such as para-sports, represent a strong alternative for achieving inclusion, offering environments where children participate on equal terms and where leaders possess the skills to recognise individual strengths and possibilities. Altogether, such approaches can help ensure that the vision of “Joy of sports—for all” (24) becomes a reality rather than just an aspiration.

In practice, achieving meaningful leisure opportunities for all children requires adopting multiple strategies that allow children to choose desired activities, regardless of disability. In the context of organised leisure, this entails a dual focus: making mainstream activities, such as sports and cultural programs, accessible and inclusive while also offering tailored options, such as para-sports. Informal activities should be equally recognised for their value, offering flexibility and autonomy that complement mainstream and adapted formats. These approaches are not mutually exclusive; rather, they should work together to ensure that all children can enjoy meaningful and inclusive leisure experiences.

## 5 Limitations

This study has several limitations that should be acknowledged. First, it relies on self-reported data, which may be subject to bias in how children interpret and report their leisure activities. Second, the “two-step” operationalisation of disability (functional limitation + participation restriction) aligns with international recommendations (e.g., the Washington Group) andwith the relational model of disability. This is considered a strength, as it improves validity compared to single-item measures and identifies children who encounter limitations in important areas of life (62). However, it still remains a broad categorisation. The influence of different types of disabilities on leisure participation, spanning physical, sensory, and cognitive dimensions, was not examined. This limits the ability to draw conclusions for targeted interventions addressing specific disability groups, as environmental barriers and facilitators can vary substantially among them. Future studies should investigate whether leisure profiles differ across specific subgroups of disabilities, as this could inform more tailored inclusion strategies and policy interventions.

A third limitation is that children were assigned to their most likely latent class (modal assignment), a common practice in applied LCA research (53, 74). This approach was selected because it provides a clear and interpretable classification of individuals, which is particularly useful for descriptive and applied purposes. Although modal assignment does not account for classification uncertainty and may attenuate associations (71, 73), the entropy value indicated moderate classification quality (entropy = 0.69), reducing the likelihood of existing bias. Nevertheless, the moderate entropy suggests some classification uncertainty (74, 77), particularly between the Aesthetic- and Screen-oriented classes and the Home- and Sport-oriented classes. This suggests that some participants may share characteristics across classes, blurring conceptual boundaries between profiles. Consequently, individuals may have similar probabilities of belonging to two classes, which introduces uncertainty when interpreting associations with factors such as disability status or sociodemographic characteristics. In practice, this overlap means that interventions designed for one profile might also need to consider another, as similarities between classes could influence targeting strategies. More advanced three-step methods (e.g., R3STEP, BCH) are recommended when feasible (71, 76), but these were not implemented here due to the exploratory nature of the analysis.

A fourth limitation is that, although model fit improved when covariates were added, the pseudo R² values indicate limited explanatory power, which is common in social research (100, 101). Lastly, the study's cross-sectional design allows for the identification of associations between variables but does not permit causal inferences (65). For example, while the findings indicate that CWD are more likely to belong to screen-oriented profiles, the directionality of this relationship remains unclear. It is not possible to determine whether disability leads to increased screen use, whether screen use exacerbates challenges faced by children with disabilities, or whether other, unmeasured factors influence both. Future longitudinal research is needed to explore these relationships over time and to better understand the causal mechanisms underlying leisure participation patterns among CWD.

## 6 Conclusion and future directions

This study highlights persistent disparities in leisure participation among CWD compared to their peers without disability, particularly their underrepresentation in sports-oriented profiles and overrepresentation in screen-dominated profiles. These patterns underscore the need to address structural and social barriers in formal activities, such as organised sports, while also recognising the potential of digital and home-based arenas as inclusive spaces for leisure engagement.

This study also has practical implications for municipalities, schools, care givers, sports organisations, and voluntary organisations. We highlight the importance of implementing targeted strategies to reduce structural and social barriers to participation, especially in more formal and organised activities. This may include universal design, inclusive leisure education practices and also active recruitment for CWD. Furthermore, tailored-made interventions may also foster inclusion and mastery. Additionally, digital leisure can be leveraged as a platform for inclusion of CWD in leisure-time activities. For example, e-sports is a rapidly growing arena that can foster mastery, teamwork, and social connection (90). Future research should explore how environmental and social factors influence participation, as well as how digital and cultural spaces can facilitate inclusion. Additionally, we suggest that researchers examine whether leisure profiles differ across specific disability subgroups (e.g., physical, sensory, cognitive).

## Data Availability

The data used in this study are not publicly available due to restricted access, in accordance with the study's approval by the Norwegian Agency for Shared Services in Education and Research and requirements of data protection legislation. All scripts will be available on dataverse.no/dataverse/usn after publication.
