# Supplementary material for: Leisure time patterns of children with and without disabilities: a cross-sectional latent class analysis
Source: Front Sports Act Living. 2026 Jan 6;7:1712055. doi: 10.3389/fspor.2025.1712055 (PMC12815810; doi:10.3389/fspor.2025.1712055)
Supplement: Supplementary file 1 [file Datasheet1.pdf]

Supplementary Material

Supplementary Table 1a: Operationalisation of the leisure variables (part 1)

	Question	Original response	Adjusted response
Home and neighbourhood leisure	How often do you do any of the following things close to where you live?<br>*Hang out on your own or with friends/siblings without doing anything special?*<br>*Play outside on your own or with friends/siblings (for example, playing football, cycling or playing tag)*	Rarely or never	Rarely or never
		A few times a month	
		About once a week	About once a week
		2–3 times a week	Between 2 and 3 times a week
		4–5 times a week	Four times a week or more
		About every day	
	When you're at home, how often do you:<br>*Have a visit from friends?*<br>*Do something nice with the adults in your family*<br>*Help with housework (for example, cooking, cleaning, washing, hoovering, gardening)?*<br>*Make something or do things with your hands (for example drawing, painting, carpentry, handiwork or 'tinkering')*	Rarely or never	Rarely or never
		A few times a month	
		About once a week	Once a week
		2–3 times a week	Between 2 and 3 times a week
		4–5 times a week	Four times a week or more
		About every day	
Digital leisure	Think about an ordinary day after school. Roughly how many hours do you spend on the following?<br>*Watch TV/films/series/YouTube*<br>*Play computer games/TV games*<br>*Play on a mobile phone/tablet*<br>*On social media*	No time	1 hour or less
		Half an hour or less	
		Between half an hour and an hour	
		1–2 hours	Between 1 and 3 hours
		2-3 hours	
		3-4 hours	3 hours or more
		4 hours or more	
Organised leisure	What leisure activities do you do, and how often do you do them? One tick for each line.<br>*Sports, Dance or ballet, Theatre, singing or music, Youth club or junior club, Other activities*	Never	Never
		Less than once a week	Less than once a week
		Once a week	Once a week
		Several times a week	Several times a week
Unorganised leisure	How often do you normally work out?	About every day	Every day
		4–5 times a week	2 - 5 times a week
		2–3 times a week	
		About once a week	Once a week or less
		A few times a month	
		Rarely or never	Never
	How often do you do outdoor activities in the summer?<br><br>How often do you do outdoor activities in the winter?	Never	Never
		Rarely	Rarely or sometimes
		Sometimes	
		Often	Often or very often
		Very often	

**Supplementary Table 1b:** Operationalisation of the leisure variables (part 2)			
	**Question**	**Original response**	**Adjusted response**
Schoolwork and Unorganised social or academic leisure	How much time do you usually spend on schoolwork during the afternoon and evening?	No time	No time
		Less than half an hour	30 min or less
		About half an hour	Between 30 min and 1 hour
		About 1 hour	
		About 2 hours	2 hours or more
		More than 2 hours	
	How often do you usually? *Go to a café, shopping centre or shops*	Rarely or never	Rarely or never
		A few times a month	
		About once a week	Once a week
		2–3 times a week	Between 2 and 3 times a week
		4–5 times a week	Four times a week or more
		About every day	
	How often do you usually? *Go to the library*	Rarely or never	Rarely or never
		A few times a month	
		About once a week	Once a week or more
		2–3 times a week	
		4–5 times a week	
		About every day	
	Think about an ordinary day after school. Roughly how many hours do you spend on? *Reading books, comics or listening to audiobooks*	No time	Never
		Between half an hour and an hour	1 hour or less
		1–2 hours	More than 1 hour
		2-3 hours	
		3-4 hours	
		4 hours or more	

Supplementary Figure 1: Classification Heatmap from LCA Analysis

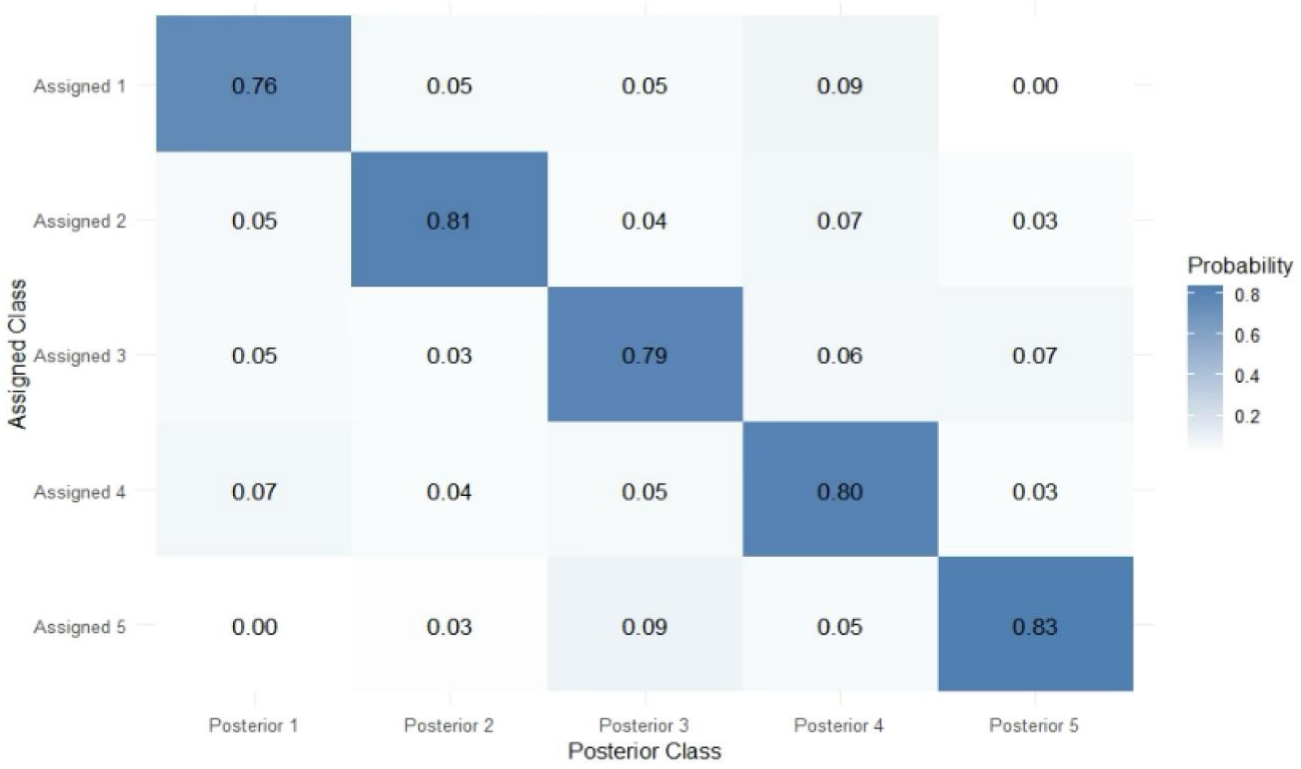

Supplementary Table 2a: Multinomial regression of latent class analysis. Class 1(The Home-oriented) as reference class. (Odds ratio with 95% confidence interval)

	**The Social-oriented (Class 2)**	**The Aesthetic-oriented (Class 3)**	**The Physically-oriented (Class 4)**	**The Screen-oriented (Class 5)**
**Model 1 (n=5538)**				
Disability status (ref. Not disabled)				
Disabled	1.98** (1.44-2.72)	1.29 (0.94-1.78)	0.93 (0.68-1.26)	2.37** (1.74-3.24)
**Model 2 (n=5479)**				
Disability status (ref. Not disabled)				
Disabled	1.89** (1.37-2.61)	1.21 (0.88-1.68)	0.93 (0.68-1.27)	2.24** (1.62-3.08)
Sex (ref. Boy)				
Girl	0.67** (0.55-0.80)	1.59** (1.34-1.89)	0.54** (0.46-0.63)	0.55** (0.54-0.66)
Grade (ref 5th)				
6th	1.31* (1.06-1.64)	1.14 (0.93-1.39)	1.59** (1.33-1.92)	1.44* (1.14-1.82)
7th	1.79** (1.41-2.26)	1.71** (1.38-2.12)	2.95** (2.42-3.59)	2.89** (2.27-3.66)
Language spoken at home (Ref. Only Norwegian)				
Norwegian and/or another language	1.50** (1.20-1.86)	1.02 (0.83-1.26)	0.74* (0.60-0.90)	1.10 (0.87-1.39)
Centrality (ref. Rural)				
Urban	1.31* (1.10-1.56)	1.07 (0.92-1.24)	1.68** (1.54-1.95)	1.44** (1.21-1.73)
SES (ref Low)				
Medium	0.95 (0.81-1.12)	0.63** (0.54-0.73)	0.81* (0.70-0.92)	0.45** (0.38-0.53)
High	1.09 (0.92-1.28)	1.06 (0.92-1.23)	0.92 (0.81-1.05)	1.08 (0.92-1.28)
* p<.05, ** p<.001				

**Supplementary Table 2b:** Multinomial regression of latent class analysis. Class 2 (The Social-oriented) as reference class. (Odds ratio with 95% confidence interval)				
	**The Home-oriented (Class 1)**	**The Aesthetic-oriented (Class 3)**	**The Physically-oriented (Class 4)**	**The Screen-oriented (Class 5)**
**Model 1 (n=5538)**				
Disability status (ref. Not disabled)				
Disabled	0.51**	0.65*	0.47**	1.20
	0.37-0.70	0.48-0.88	0.35-0.62	0.89-1.61
**Model 2 (n=5479)**				
Disability status (ref. Not disabled)				
Disabled	0.53**	0.64*	0.49**	1.18
	0.38-0.73	0.47-0.87	0.37-0.66	0.87-1.60
Sex (ref. Boy)				
Girl	1.50**	2.38**	0.81*	0.82
	1.24-1.80	1.97-2.88	0.68-0.96	0.67-1.10
Grade (ref 5th)				
6th	0.76*	0.86	1.21	1.09
	0.64-0.94	0.60-1.08	0.98-1.49	0.85-1.41
7th	0.56**	0.96	1.65**	1.61**
	0.44-0.71	0.76-1.12	1.33-2.04	1.25-2.07
Language spoken at home (Ref. Only Norwegian)				
Norwegian and/or another language	0.67**	0.68**	0.49**	0.74*
	0.54-0.83	0.55-0.85	0.40-0.60	0.58-0.93
Centrality (Rural)				
Urban	0.76*	0.81*	1.28*	1.10
	0.64-0.91	0.68-0.97	1.28-1.52	0.90-1.34
SES (ref Low)				
Medium	1.04	0.66**	0.85*	0.45**
	0.89-1.23	0.56-0.78	0.73-0.98	0.40-0.57
High	0.92	0.98	0.85*	1.00
	0.78-1.08	0.83-1.15	0.73-0.98	0.83-1.19
* p<.05, ** p<.001				

Supplementary Table 2c: Multinomial regression of latent class analysis. Class 3 (The Aesthetic-oriented) as reference class. (Odds ratio with 95% confidence interval)

	**The Home-oriented (Class 1)**	**The Social-oriented (Class 2)**	**The Physically-oriented (Class 4)**	**The Screen-oriented (Class 5)**
**Model 1 (n=5538)**				
Disability status (ref. Not disabled)				
Disabled	0.78	1.53*	0.72*	1.84**
	0.56-1.07	1.13-2.08	0.54-0.96	1.37-2.47
**Model 2 (n=5479)**				
Disability status (ref. Not disabled)				
Disabled	0.83	1.56**	0.77	1.85**
	0.60-1.14	1.15-2.13	0.57-1.03	1.36-2.50
Sex (ref. Boy)				
Girl	0.63**	0.42**	0.34**	0.34**
	0.53-0.75	0.35-0.51	0.29-0.40	0.28-0.42
Grade (ref 5th)				
6th	0.88	1.16	1.40**	1.25
	0.72-1.07	0.92-1.45	1.16-1.70	1.00-1.60
7th	0.58**	1.05	1.72**	1.69**
	0.47-0.72	0.83-1.32	1.42-2.09	1.34-2.13
Language spoken at home (Ref. Norwegian)				
Norwegian and/or another language	0.98	1.47**	0.72**	1.08
	0.79-1.09	1.18-1.82	0.59-0.88	0.86-1.36
Centrality (Rural)				
Urban	0.94	1.23*	1.47**	1.35**
	0.81-1.09	1.03-1.47	1.35-1.83	1.13-1.62
SES (ref Low)				
Medium	1.59**	1.52**	1.28**	0.72**
	1.47-1.84	1.29-1.78	1.12-1.47	0.61-0.85
High	0.94	1.02	0.87*	1.02
	0.81-1.09	0.87-1.21	0.76-0.99	0.86-1.21

* p<.05, ** p<.001

**Supplementary Table 2d:** Multinomial regression of latent class analysis. Class 5(The Screen-oriented) as reference class. (Odds ratio with 95% confidence interval)				
	**The Home-oriented (Class 1)**	**The Social-oriented (Class 2)**	**The Aesthetic-oriented (Class 3)**	**The Physically-oriented (Class 4)**
**Model 1 (n=5538)**				
Disability status (ref. Not disabled)				
Disabled	0.42**	0.83	0.54**	0.39**
	0.31-0.58	0.62-1.12	0.40-0.73	0.29-0.52
**Model 2 (n=5479)**				
Disability status (ref. Not disabled)				
Disabled	0.45**	0.85	0.54**	0.42**
	0.32-0.62	0.63-1.15	0.40-0.74	0.31-0.55
Sex (ref. Boy)				
Girl	1.82**	1.22	2.90**	0.99
	1.50-2.21	0.99-1.49	2.39-3.52	0.83-1.18
Grade (ref 5th)				
6th	0.70*	0.92	0.79	1.11
	0.55-0.88	0.71-1.18	0.62-1.00	0.88-1.39
7th	0.35**	0.62**	0.59**	1.02
	0.27-0.44	0.48-0.80	0.47-0.75	0.82-1.27
Language spoken at home (Ref. Only Norwegian)				
Norwegian and/or another language	0.91	1.36*	0.93	0.67**
	0.72-1.14	1.08-1.71	0.47-1.16	0.64-0.83
Centrality (Rural)				
Urban	0.69*	0.91	0.74**	1.16
	0.58-0.83	0.74-1.11	0.62-0.89	0.98-1.39
SES (ref Low)				
Medium	2.22**	2.11**	1.39**	1.79**
	1.87-2.62	1.77-2.52	1.18-1.65	1.53-2.09
High	0.92	1.00	0.98	0.85*
	0.78-1.09	0.84-1.20	0.83-1.16	0.73-0.99
* p<.05, ** p<.001				
